# Patient Reported Experience Measure in Endoscopic Ultrasonography: The PREUS Study Protocol

**DOI:** 10.3390/nursrep12010007

**Published:** 2022-02-08

**Authors:** Laura Apadula, Gabriele Capurso, Alessandro Ambrosi, Paolo Giorgio Arcidiacono

**Affiliations:** 1 Pancreatobiliary Endoscopy and Endosonography Division IRCCS San Raffaele, Pancreas Translational and Clinical Research Center, IRCCS San Raffaele, Via Olgettina 60, 20132 Milan, Italy; apadula.laura@hsr.it (L.A.); arcidiacono.paologiorgio@hsr.it (P.G.A.); 2 Surgery and Medicine Department, Vita-Salute San Raffaele University, Via Olgettina 58, 20132 Milan, Italy; ambrosi.alessandro@hsr.it

**Keywords:** PREM, patient experience, patient-reported experience measure, endoscopic ultrasonography, pancreatobiliary endoscopy

## Abstract

The evaluation of the patient’s experience is becoming increasingly important as a better patient experience can improve the quality of the health service delivered. Patient-reported experience measures (PREMs) are self-report assessment tools provided to patients about their experience during any health event. There are few PREM instruments in the field of gastrointestinal endoscopy, and none is specific for endoscopic ultrasound (EUS). This study aims to develop a questionnaire to evaluate the experience of patients undergoing EUS, identifying and prioritizing the factors related to the patient’s experience. The study will consist of several phases: (A) tool creation; (B) face and content validity; (C) ranking: to evaluate the relevance of the identified questions in the previous phase; (D) questionnaire creation and validity testing. The final output will be the production of a specific tool that can be used to measure patients’ experience during EUS. This questionnaire may become a relevant part of actions taken to measure the quality of care provided to patients undergoing EUS. Furthermore, correlation between health care providers’ and patients’ views of the relevance of the included items will allow optimization of empathetic and psychological aspects.

## 1. Introduction

Patient centeredness is considered to be an important feature of high-quality patient care [1,2] and, consequently, the evaluation of the patient’s experience is becoming increasingly important in modern medicine [3,4,5,6]. There is increasing focus on investigating how a better patient experience can improve the quality of the health service offered [7,8,9,10]. The process of ensuring continuous improvement in the quality of services begins with an attempt to define which aspects of care have an impact on the patient’s experience [11]. Usually, this aspect is investigated through the use of questionnaires [12]. However, most tools of this kind are constructed by physicians without patient involvement [5,13,14,15].

A more appropriate approach includes questioning the patient about their needs and wishes regarding the care process [5,7,16]. Patient-reported experience measures (PREMs) are self-report assessment tools provided to patients about their experience during any health event, in order to inform health care professionals (HCP) about how the event was experienced [5]. The added value of a self-report measure is that the patient offers a view of the event that is complementary to the physician’s, providing information on the humaneness of the care [12]. Several studies have shown that there is considerable disparity between the perception of patients and that of HCPs [17,18,19,20]. In the specific field of gastrointestinal endoscopy (GIE), there are few instruments dedicated to this task [21], and they usually concern upper/lower gastrointestinal (GI) tract endoscopy procedures [22,23,24,25].

Improving the quality of GIE has become increasingly important [26,27,28,29,30] and this has involved measuring patient satisfaction, but GI-endoscopy-PREMs are limited, and most are not derived from patients themselves [7,8,17].

In the more specific area of pancreato-biliary endoscopy, PREMs for endoscopic ultrasonography (EUS) and endoscopic retrograde cholangiopancreatography (ERCP) have not been established and data are scattered and heterogeneous [21,30,31]. Since EUS and ERCP are amongst the most advanced and complex endoscopic procedures [26,27,32], the creation and validation of patient-reported indicators should be a priority to monitor both endoscopist and endoscopy service performance [21,33,34].

The present study protocol is focused on EUS only, and not on ERCP, for two different reasons: (1) “Hospitalization bias”: patients who undergo ERCP are often hospitalized. It is recognized that hospitalization per se can have either a negative or positive impact on the patient’s experience [20,35] and that this might create a bias regarding the experience related only to the diagnostic procedure itself. However, as most diagnostic EUS are performed in an outpatient setting, a specific tool is needed to evaluate a number of typical aspects of patient reported experience, such as waiting times for examinations, that are relevant to the patient’s experience [17]; (2) More representative sample: EUS is performed on a larger set of disorders (both malignant and benign), with a greater variability of patients (both elderly and younger patients), creating the possibility for data stratification in various types of patients/conditions and, consequently, a more complete and accurate description of the patient’s experience. The purpose of this study is to develop a tool (questionnaire) to evaluate the experience of outpatients who undergo EUS, identifying and prioritizing the factors related to the patient’s experience. We aim to highlight the viewpoint of patients and health care professionals (HCP), in order to be able to ensure an integrated and complementary view.

*Primary outcome:* develop a questionnaire to measure patient reported experience during EUS (PREUS).

*Secondary outcome:* identify aspects of care that are most relevant, both for patients undergoing EUS, and HCPs involved in this procedure.

## 2. Materials and Methods

### 2.1. Study Workflow and Data Collection and Analysis

This will be a monocentric observational study consisting of several phases (summarized in Table 1):

A. Tool creation: Based on a previous systematic literature review [21], a draft of a tool containing questions about patients’ experience during EUS procedures will be created.
B. Face and content validity: the initially developed tool will undergo two different rounds of expert evaluation in this step. In the first round, the tool will be evaluated by a panel of stakeholders (experts’ opinion) involved in the care of patients with pancreaticobiliary disorders (two endoscopists and two nurses with experience in bilio-pancreatic disorders and EUS, one psychologist with expertise in patients’ engagement, one expert in the philosophy of communication and a representative of a pancreatic malignancies patients’ advocacy group). The face and content validity of the instrument and the content validity index (CVI) will be calculated. An explicit description of domains that comprise the preliminary draft of the tool will be sent to the involved experts to check if any important area is missing, and/or to receive suggestions regarding additional items to fill these gaps, and to rate the importance of the questions [36]. Based on these results, a new draft of the questionnaire will be submitted to a set of at least 30 outpatients undergoing EUS, who already underwent this procedure at least once (expert patients’ opinion). EUS procedures will be performed under deep sedation with an intravenous infusion of Propofol (Diprivan^®^, Zeneca, Germany) [37]. The CVI will again be calculated, as well as the internal consistency evaluated by means of Cronbach’s alpha. Questions with a value < 0.90 will be excluded and a final draft of the questionnaire will be created.
C. Ranking: the questionnaire obtained after the previous steps will be evaluated both by 50 outpatients undergoing EUS who have signed the informed consent and by 21 health care professionals (HCPs) (7 endoscopists and 14 endoscopy nurses) involved in the same procedure, to rate each question’s relevance on a continuous Likert scale (0–10). Differences in the ranking of questions between HCPs and patients and the possible correlation coefficients between the mean values of the scale for each question will be investigated (Spearman correlation). HCPs and patients will rank each individual question concerning aspects of the endoscopic experience with a score from 1 to 10 (1 = less important; 5 = relatively important; 10 = more important) in terms of how each aspect influences their experience with EUS procedures. The same tool and scale will be submitted to the HCPs involved with EUS in the local endoscopy unit after signing the informed consent; HCPs will rank the items twice: (1) they will fill-in a first tool based on what they think would most influence patients’ EUS experience; (2) they will fill-in a second, identical, tool considering what is important from the patient’s viewpoint, as if they were a patient.
  We will also collect data about age, gender, family status, hometown, residence, instruction level, and, for HCPs, the professional category (doctor or nurse), to determine their correlation with the results. As the study will be conducted during the COVID-19 pandemic, a specific question on how worried patients are about having to come to the hospital will also be included. The whole questionnaire will be filled-in anonymously in paper form and left in a box at the service exit. Finally, we will analyze the results of all respondents to compare patient and HCP views on EUS experience and create a ranking of the most important questions based on the results for both tools.
D. Questionnaire creation and validity testing: based on the results of the previous phases, we will develop a final version of the questionnaire (PREUS) and test it in a monocentric study. To this end, the questionnaire will be administered to 100 consecutive patients undergoing EUS, with informed consent, to obtain information on compliance. Patients will be asked to rank each individual question concerning aspects of their experience of the EUS procedure with a score from 1 to 5 (1 = poor; 3 = good; 5 = excellent experience). Again, data on age, gender, family status, hometown, residence, instruction level and worries about COVID-19 will be recorded, in order to investigate any possible correlation with the results. For this last phase, the questionnaire will be filled in anonymously on a tablet on a web-based platform created and dedicated to the analysis of PREMs (Brightfish BV, The Netherlands) with the support of a research nurse or other properly trained staff. If any type of difficulty arises (e.g., tablet not available, web-platform error or direct request by a patient), the questionnaire will be completed in paper form and left in a dedicated box at the service exit. Exploratory factor analysis (EFA) and varimax rotation to assess the validity of the instrument will be performed.

### 2.2. Inclusion Criteria for Enrolled Patients

The following inclusion criteria have been set

- Age > 18 years;
- Outpatients undergoing EUS in the local endoscopy unit;
- Written informed consent signed.

## 3. Expected Results and Discussion

The present study protocol is aimed at developing a novel, specific tool to measure patients’ experience during EUS. This questionnaire may become a relevant part of actions taken to measure the quality of care provided to patients undergoing EUS. Furthermore, a correlation between health care providers’ and patients’ views of the relevance of the included items will allow optimization of empathetic and psychological aspects. In this regard, we hypothesize that there may be a higher correlation between the patients’ and nurses’ views compared to that between patients’ and physicians’ views [38].

## Figures and Tables

**Table 1:** Schematic representation of the study steps toward the creation of a dedicated tool to measure patients’ reported experience during endoscopic ultrasound (EUS).

Step	Objective
(A) Tool creation	- Create draft “patient reported measure of EUS” (PREUS) questionnaire based on literature review.
(B) Face and Content Validity	- Submit “PREUS” to 7 experts in the field and to “expert” patients. - Calculate content validity index (CVI). - Modify accordingly.
(C) Ranking	- Evaluate the relevance of items for 50 outpatients undergoing EUS (scale from 1 to 10). - Evaluate the relevance of questions by health care professionals (HCPs) to measure the agreement with patients. - Calculate Cronbach’s alpha. - Create a final draft of the questionnaire.
(D) Questionnaire administration and validity testing	- Submit “PREUS” to a further 100 consecutive outpatients undergoing EUS to test compliance and validity.

## Data Availability

Not applicable.
